# High-throughput and high-resolution powder X-ray diffractometer consisting of six sets of 2D CdTe detectors with variable sample-to-detector distance and innovative automation system

**DOI:** 10.1107/S1600577524003539

**Published:** 2024-06-20

**Authors:** Shogo Kawaguchi, Shintaro Kobayashi, Hiroki Yamada, Hirotaka Ashitani, Michitaka Takemoto, Yasuhiko Imai, Takaki Hatsui, Kunihisa Sugimoto, Osami Sakata

**Affiliations:** a Japan Synchrotron Radiation Research Institute (JASRI) 1-1-1 Kouto Sayo-cho, Sayo-gun Hyogo679-5198 Japan; b RIKEN SPring-8 Center, 1-1-1 Kouto, Sayo-cho, Sayo-gun, Hyogo679-5148, Japan; ESRF – The European Synchrotron, France

**Keywords:** powder X-ray diffraction, high-throughput devices, high-energy X-rays, millisecond time-resolved experiments

## Abstract

The demand for powder X-ray diffraction analysis continues to increase in a variety of scientific fields, as the excellent beam quality of high-brightness synchrotron light sources enables the acquisition of high-quality measurement data with high intensity and angular resolution. Synchrotron powder diffraction has enabled the rapid measurement of many samples and various *in situ*/*operando* experiments in nonambient sample environments. To meet the demands for even higher throughput measurements using high-energy X-rays at SPring-8, a high-throughput and high-resolution powder diffraction system has been developed. This system is combined with six sets of two-dimensional (2D) CdTe detectors for high-energy X-rays, and various automation systems, including a system for automatic switching among large sample environmental equipment, have been developed in the third experimental hutch of the insertion device beamline BL13XU at SPring-8. In this diffractometer system, high-brilliance and high-energy X-rays ranging from 16 to 72 keV are available. The powder diffraction data measured under ambient and various nonambient conditions can be analysed using Rietveld refinement and the pair distribution function. Using the 2D CdTe detectors with variable sample-to-detector distance, three types of scan modes have been established: standard, single-step and high-resolution. A major feature is the ability to measure a whole powder pattern with millisecond resolution. Equally important, this system can measure powder diffraction data with high *Q* exceeding 30 Å^−1^ within several tens of seconds. This capability is expected to contribute significantly to new research avenues using machine learning and artificial intelligence by utilizing the large amount of data obtained from high-throughput measurements.

## Abstract 2

A high-throughput and high-resolution powder X-ray diffractometer has been developed in the third experimental hutch of BL13XU, SPring-8. The diffractometer is equipped with six sets of 2D CdTe detectors and an automation system including sample exchange and equipment switching. Performance and demonstration results are presented.

## 1. Introduction

The role of powder X-ray diffraction in materials characterization remains unparalleled, emphasizing its foundational status in materials science and engineering. The capabilities of powder diffraction when coupled with synchrotron sources have been demonstrated to be expansive, providing high angular and temporal resolutions across a wide *Q* range in markedly reduced time frames. Therefore, various high-energy powder X-ray diffraction beamlines have been constructed at synchrotron radiation facilities worldwide to meet the great demand in the materials science and engineering fields over the past few decades. Recently, many scientific disciplines, including the energy and environmental fields, have stimulated the need to measure many samples for systematic characterization and clarification of their behaviour using powder and bulk samples under actual operating conditions.

At the end of the 20th century, the operation and public use of the powder diffraction system on the bending magnet beamline BL02B2 began at the third-generation synchrotron facility SPring-8 in Japan, and the beamline has contributed to the materials research of many users. In the experimental hutch, a large Debye–Scherrer camera with a two-dimensional (2D) imaging-plate detector was developed for accurate powder diffraction data collection (Nishibori *et al.*, 2001 ▸). This instrument has enabled studies of the maximum-entropy method charge densities of a wide range of materials (Kuroiwa *et al.*, 2001 ▸; Kitaura *et al.*, 2002 ▸; Nishibori *et al.*, 2007 ▸). In the 2010s, to meet the need for *in situ* measurements performed while changing the sample conditions, multiple modular one-dimensional (1D) Si microstrip MYTHEN (Schmitt *et al.*, 2003 ▸) detectors were introduced to the powder diffractometer. Powder diffractometers equipped with MYTHEN or MYTHEN-II detectors have been widely installed on powder diffraction beamlines (Haverkamp & Wallwork, 2009 ▸; Bergamaschi *et al.*, 2010 ▸; Thompson *et al.*, 2011 ▸; Fauth *et al.*, 2013 ▸; Carvalho *et al.*, 2016 ▸; Kawaguchi *et al.*, 2017 ▸; Osaka *et al.*, 2019 ▸). These 1D detector systems have lower angular resolution than scintillation detector systems combined with analyser crystals (Fitch, 2004 ▸; Thompson *et al.*, 2009 ▸; Schökel *et al.*, 2021 ▸) but they can quickly collect a whole powder pattern with pragmatic angular resolution, which is usually higher than that obtained with area detectors, and systems with photon-counting readouts can cover a wide angular range and have a high dynamic range.

The benefits of the 1D detectors have facilitated new possibilities for *in situ* experiments on BL02B2, such as gas separation/adsorption/desorption measurements (Hiraide *et al.*, 2020 ▸; Su *et al.*, 2022 ▸), negative thermal expansion measurements (Nishikubo *et al.*, 2019 ▸), solid-state ceramic synthesis (Miura *et al.*, 2021 ▸), and high-throughput characterization of nanoalloys (Kusada *et al.*, 2021 ▸), piezoelectric ceramics (Kuroiwa *et al.*, 2020 ▸) and thermoelectric materials (Oku *et al.*, 2021 ▸). To meet the further diverse requirements of the academic and industrial sectors, with the objective of analysing functional materials under various sample environments, the following issues must be addressed: (i) the *Q* range (where *Q* = 4πsinθ/λ) needs to be wider than 20 Å^−1^ to allow partially disordered materials to be investigated, (ii) the measurement time needs to be shorter than a second for rapid *in situ* and *operando* experiments, (iii) to accelerate high-throughput measurements, a highly automated measurement system must be able to acquire large amounts of diffraction data within a short period for systematic sample characterization, with the potential use of machine learning, and (iv) a large sample space is needed to install carry-in equipment to achieve a variety of *in situ* observations. Issues (i) and (ii) are difficult to solve on a bending-magnet source beamline. In addition, to exploit high-energy X-rays, the use of a high-*Z* material sensor is indispensable because the detection efficiency of an X-ray detector with an Si sensor decreases when using high-energy X-rays. For example, in the case of a 1 mm thick Si sensor, the efficiency decreases to less than 20% for 35 keV X-rays. Recently, with the use of high-energy X-rays, a unique setup equipped with a 2D hybrid pixel array with CdTe sensors (such as PILATUS3 or EIGER2, Dectris Ltd) and flat-panel detectors (FPDs) has been introduced [see *e.g.* Dippel *et al.* (2015 ▸), Chater *et al.* (2017 ▸), Dejoie *et al.* (2018 ▸) and Fitch *et al.* (2023 ▸)]. These developments on insertion device (ID) beamlines have provided excellent data with enhanced angular resolution and a wide *Q* range, and have improved the statistical quality of high-angle data.

Issues (iii) and (iv) regarding BL02B2 are closely related to the automation systems. High-throughput data collection in powder X-ray diffraction is crucial for screening and characterizing large amounts of material. Therefore, automatic sample exchange systems, such as robotic sample changers (SCs), have been installed on beamlines worldwide. On the BL02B2 beamline at SPring-8, an automated SC for 50 samples and an automatic sample positioning system for capillary samples have been incorporated (Kawaguchi *et al.*, 2017 ▸). These systems not only optimize beam-time utilization but also reduce the workload for users. However, as described above, there are many requirements for the various sample environments on powder diffraction beamlines. Hence, when the measurement equipment is changed on BL02B2, the automatic SC needs to be installed/removed to secure the sample space around the diffractometer, especially for *in situ* and *operando* measurements that require a large sample environment. The installation/removal of these types of equipment and adjustment of their positions for powder X-ray diffraction measurements take a considerable amount of time and effort. In addition, the setup for the sample environments may be changed multiple times, even during one day of a user’s beam time; efficient and reproducible device switching is required to make effective use of beam time and enable flexible experimental plans for users. Therefore, we have incorporated an automated system that allows a wide range of sample environments and includes automatic sample exchange.

Herein, we introduce a new high-resolution powder diffractometer installed on the BL13XU ID beamline. This diffractometer is equipped with six sets of 2D CdTe detectors (LAMBDA 750k, X-Spectrum GmbH) to achieve high-throughput measurements. The instrument addresses the challenges mentioned above and provides enhancements in angular resolution, measurable *Q* range and temporal resolution compared to those on BL02B2. Remarkably, the system can measure powder diffraction patterns featuring high angular resolution within milliseconds. It is adept at utilizing high-energy X-rays up to 72 keV. The target materials for this measurement system span from crystalline and disordered crystalline matter to nanoparticles. Its applications are diverse, ranging from qualitative analysis of powder samples to in-depth studies of structural properties. These properties are vital in understanding physical attributes, and our system facilitates this understanding through techniques such as Rietveld analysis and pair distribution function (PDF) analysis. An advanced automation system is incorporated, and the equipment switching system provides a spacious sample area of approximately 800 mm × 600 mm, facilitating various *in situ* and *operando*X-ray diffraction experiments.

## 2. Development of the apparatus and configuration

### 2.1. Brief description of the beamline

A new powder diffraction measurement system was installed in the third experimental hutch of the X-ray diffraction and scattering I beamline (BL13XU). The light source of BL13XU is the standard-type SPring-8 in-vacuum undulator with a 32 mm period. A double-crystal monochromator with Si(111) and Si(311) crystals is positioned 50 m from the light source, and the crystals are cooled by liquid nitro­gen. A photon energy ranging from 5 to 72 keV is available. The Si(111) and Si(311) crystals are horizontally installed side by side and the crystal plane can be switched via horizontal translation. To reject higher harmonics of incident photons, two flat mirrors 0.7 m in length with two stripes of a Pt coating and an Si surface are located 54 and 55 m from the light source. By bending the mirror, an X-ray beam focused down to approximately 100 µm in the horizontal direction can be used.

**Figure 1:**
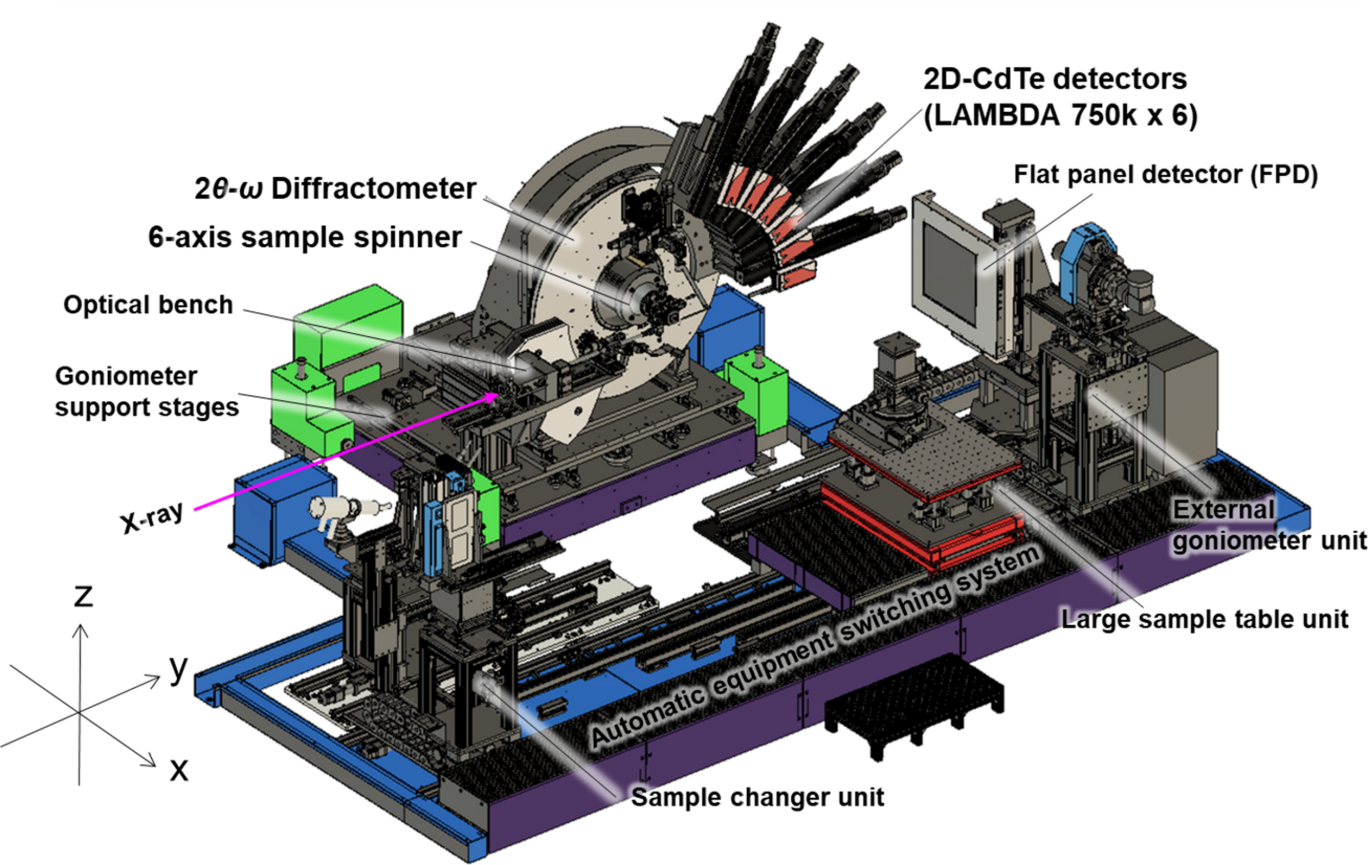
A schematic illustration of the developed high-resolution powder diffraction system in the third experimental hutch of beamline BL13XU.

An overall schematic illustration of the developed measurement system is shown in Fig. 1 ▸. The system is composed of a high-resolution powder diffractometer and an automatic equipment switching unit, as described in the following sections. The powder diffractometer is located approximately 70 m from the light source. In this instrument, X-ray energies between 16 and 72 keV are available, and a flux of approximately 10^13^ photons s^−1^ can be obtained with the Si(111) double-crystal monochromator. For X-ray energies above 37 keV, the Si(311) double-crystal monochromator can be used to obtain X-rays with a photon flux of the order of approximately 10^10^–10^11^ photons s^−1^. The photon flux in the experimental hutch was measured using a photodiode detector, and the X-ray beam was collimated by 0.65 mm (V) × 1.0 mm (H) slits. The overall information on BL13XU, including details of the optics and instruments used in the other hutches, will be reported soon. The beam size at the sample position is approximately 1 mm × 1 mm, but it is usually collimated to a size of 0.5 mm × 0.5 mm using a slit and a collimator. Most of the instrumental control and data processing at the endstation are performed using in-house *LabVIEW* software and Python scripts. Communication with various detectors and data processing systems can be controlled by connecting the main beamline computer to dedicated servers of the LAMBDA 750k detectors, an FPD and the system for 1D integration of 2D images using the *REST* API. Motor stages and other devices communicate via socket communication, and most of the GUI for experimental operation is produced in the *LabVIEW* program, which allows all experiments to be performed on a single computer.

### 2.2. High-resolution powder diffractometer

**Figure 2:**
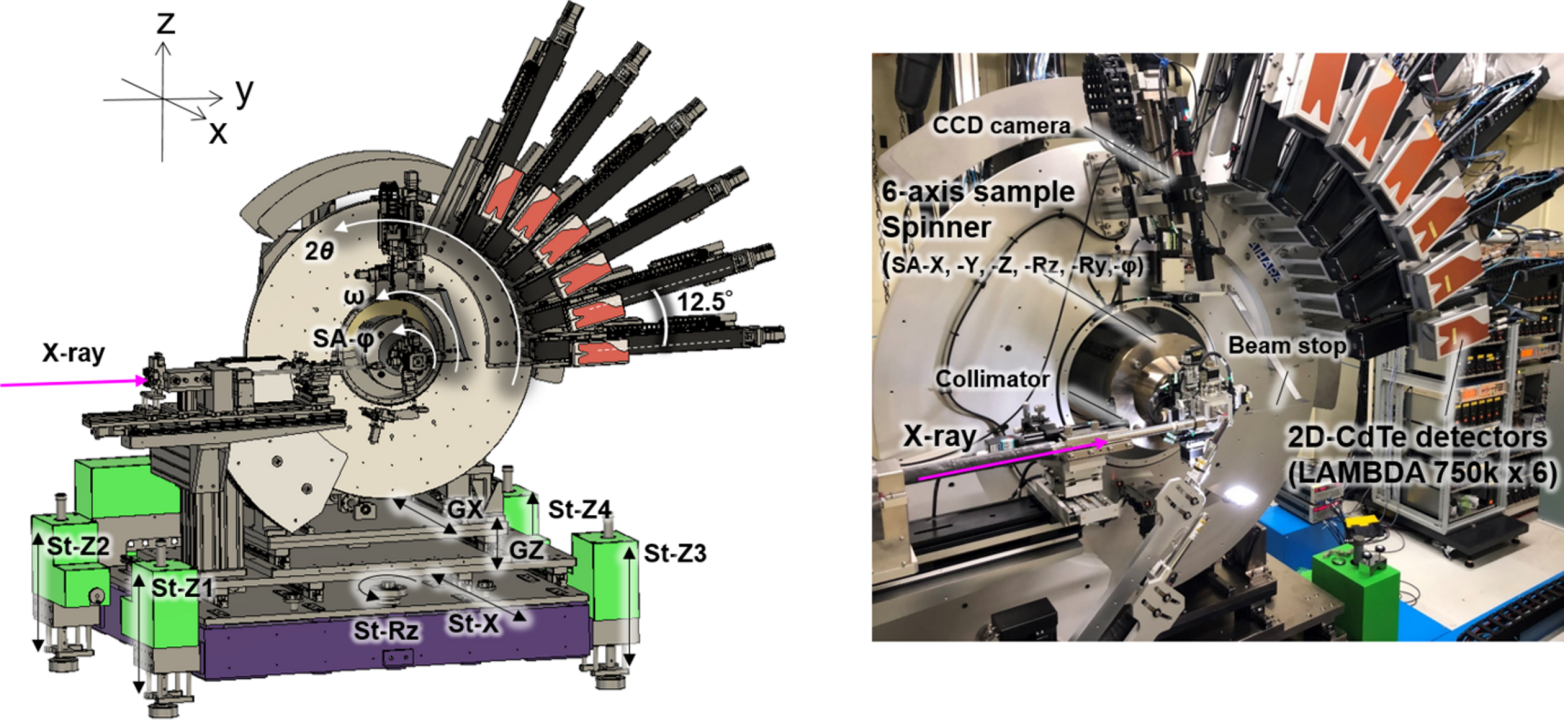
A schematic diagram and photograph of the high-resolution powder diffractometer equipped with six sets of 2D CdTe (LAMBDA 750k) detectors.

The high-resolution powder diffractometer is shown in Fig. 2 ▸. The optical bench is equipped with an ion chamber to measure the intensity of the incident X-rays, an X-ray shutter, a 4D slit and a collimator installed at the most upstream position in the diffractometer. The goniometer and optical bench are set on the support stage with an *X* translation stage (St-*X*), four independent *Z* translation stages (St-*Z*1, -*Z*2, -*Z*3 and -*Z*4), and an *Rz* rotation stage (St-*Rz*), as shown in Fig. 2 ▸; these stages are used to adjust the sample to the appropriate position and ensure parallel alignment with the X-ray beam. These stages without encoders are intended for rough adjustment of the powder diffractometer. Precise diffractometer positioning is accomplished with goniometer-*Z* (*GZ*) and goniometer-*X* (*GX*) stages. The St-*X* and St-*Z* stages can travel distances of 400 mm and 150 mm, respectively, accommodating large equipment for *operando* experiments. In addition to the St-*X* stage, the motorized *GX* and *GZ* stages are used for positioning the goniometer with an accuracy of a few micrometres. The goniometer consists of ω-axis and 2θ-axis rotation stages (440 and 480, Huber Diffraktionstechnik GmbH & Co.), both of which are equipped with rotary encoders. A motorized six-axis (SA-*X*, SA-*Y*, SA-*Z*, SA-*Ry*, SA-*Rz*, SA-φ) sample spinner is mounted on the ω axis for alignment of capillary samples. The SA-*Y* and SA-*Z* translation stages (Montblanc series, Kohzu Precision Co. Ltd), without encoders due to wiring, as well as the SA-*Ry* and SA-*Rz* tilt stages, are used for aligning the capillary samples. The SA-φ axis is equipped with a rotary encoder. The repetitive positioning accuracies of the translation stages (SA-*X*, SA-*Y*, SA-*Z*) and tilt stages (SA-*Ry*, SA-*Rz*) are less than 1 µm and 0.001°, respectively. The capillary sample can be rotated at a speed of 200 rpm by a servomotor about the φ axis during the measurement. We assumed an acquisition time of a few seconds for a typical measurement. Therefore, we considered 60 rpm or higher to be an appropriate speed. To obtain a uniform Debye ring intensity, rotation at higher speeds is better. Finally, we tested the speed and decided on a maximum of 200 rpm in view of the balance of the weight of the stage mounted on the φ axis. The eccentricity of the spinner stage is less than ±5 µm. The mounting area of the sample spinner is 70 mm^2^ and it can move approximately 300 mm along the −*X* direction using the motorized stage. Typically, powder samples packed into thin-walled glass capillaries are mounted on the spinner on the axis of the powder diffractometer. Additionally, small sample cells, such as battery cells or Linkam temperature-control stages, can also be attached to the spinner, enabling alignment of the sample cell position using the SA-*X*, SA-*Y*, SA-*Z*, SA-*Ry* and SA-*Rz* stages and *in situ* experiments with oscillation of the ω axis. Almost all of the equipment is specially designed and manufactured in collaboration with Rigaku Aihara Seiki Co. Ltd. Some of the equipment utilizes commercially available motorized stages that have been modified or incorporated into the equipment.

To accommodate multiple sets of 2D detectors on the 2θ axis, our newly developed diffractometer is larger than our previous diffractometer on BL02B2. The 2θ axis can move at speeds exceeding 1° s^−1^, and a large disc with a diameter of 1200 mm is mounted on this axis. The disc is equipped with six motorized arms containing 2D CdTe detectors, a 250 kg lead counterbalance to the detectors and a CCD camera with *XYZ* stages for sample observation. For the detector arm, due to wiring and spatial considerations, translation stages without encoders and with a positioning reproducibility of a few micrometres or less are employed. We tested the positioning reproducibility using a laser displacement meter, and the results showed that the deviation was within a few micrometres. A misalignment of a few micrometres for sample-to-detector distances of approximately 600–1100 mm has a very small effect. Indeed, the diffraction data changed negligibly after changing the sample-to-detector distance. The 2D CdTe detector (LAMBDA 750k, X-Spectrum GmbH) is a photon-counting hybrid pixel detector based on the Medipix3 technology and is a commercialized version of the previously reported one (Pennicard *et al.*, 2014 ▸). The LAMBDA 750k detector active area is 84.6 mm × 28.2 mm (1528 × 512 pixels) and the CdTe sensor thickness is 1 mm. Its main features are as follows: (i) a small pixel size of 55 µm and a high dynamic range of 24 bits, contributing to the high angular resolution and high counting statistics in powder diffraction, (ii) high-*Z* sensors for efficient detection of higher X-ray energies (95% efficiency at 60 keV) and (iii) a high frame rate of 2 kHz with a couple of tens of nanoseconds between images (Pennicard & Schmehr, 2024 ▸), which is negligibly small compared with the exposure time of 0.5 ms per frame, leading to a time-resolved X-ray diffraction experiment on the order of a millisecond. All the data in this manuscript were measured in charge summing mode with 24-bit depth. The distance between the sample and the 2D CdTe detectors can be adjusted between 480 and 1100 mm, accommodating various measurement conditions. The angular interval between the centres of the adjacent 2D CdTe detectors is 12.5°. The scanning methods using the six sets of detectors are detailed in Section 3.1.

### 2.3. Automatic equipment switching system

**Figure 3:**
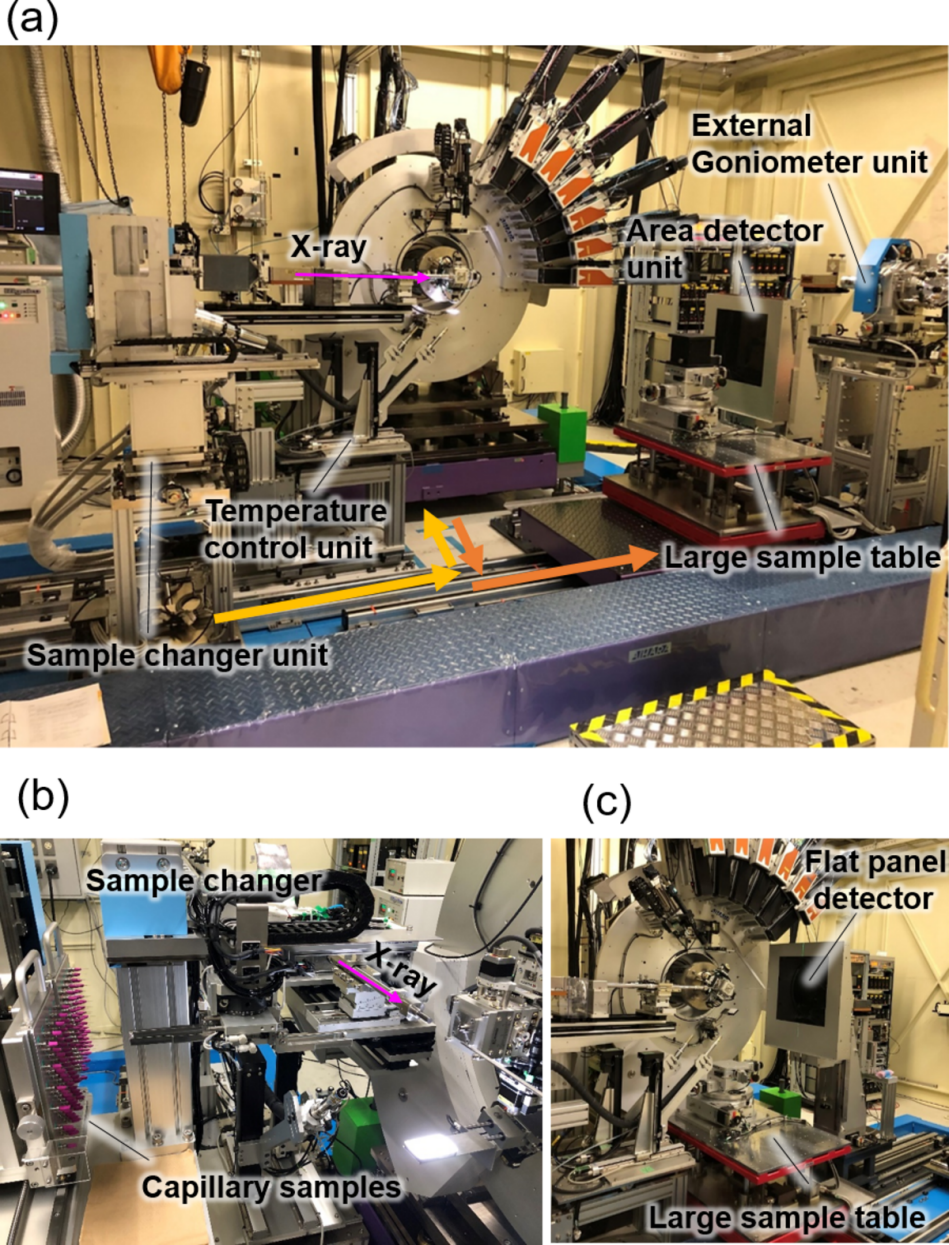
Photographs of the equipment. (*a*) The high-resolution powder diffraction system equipped with the automation system. This configuration enables a change from a large sample table to a sample changer. (*b*) The sample changer for 100 capillary samples. (*c*) The experimental setup combined with an area detector and a large sample table.

On a powder diffraction beamline, changing the measurement setup around the diffractometer is often necessary. To ensure easily reproducible reinstallation and to reduce the heavy workload of equipment switching, we developed an automatic equipment switching system, as shown in Fig. 3 ▸(*a*). The system consists of an SC, a large sample table, an external goniometer (EG), a large detector and temperature control units. The SC is an improved version of the one previously developed on BL02B2 (Kawaguchi *et al.*, 2017 ▸). Specifically, the SC has space for two magazines that can hold 50 capillary samples each, enabling 100 samples to be mounted, as shown in Fig. 3 ▸(*b*). As on BL02B2, the capillary sample position is automatically tuned with the six-axis sample spinner on the diffractometer via image recognition using the CCD camera. The large sample table has a mounting stage surface of 800 mm × 600 mm and can support objects weighing up to 500 kg. It can accommodate various large-scale equipment, such as synthetic devices, ball mills and heating furnaces. The support stage of the large sample table features extended translation stages, with translation distances of more than 200 mm for the *Z*-stage and more than 500 mm for the *Y*-stage. Typically, a horizontal rotation stage and *X*-, *Y*- and *Z*-stages are mounted on the large sample table; these stages can be easily set and removed for fine alignment of certain carried-in items. The EG stage has a vertical rotation stage for mounting heavy external equipment, such as closed-type cryostats and furnaces. The EG can be used to oscillate heavy equipment during powder diffraction measurements to improve the particle statistics. The speed of oscillation about the ω axis of the EG is approximately 5 s per degree. The three units of the SC, EG and large sample table have dedicated linear guide rails and motorized stages installed up to the front of the diffractometer, enabling accurate switching via motorized stepping motors. Each unit can travel 1950 mm in the *Y* direction and from 300 to 600 mm in the *X* direction. To switch from the large sample table to the SC, as indicated by the arrows in Fig. 3 ▸(*a*), the large sample table is first retracted, followed by insertion of the SC in front of the diffractometer. The positioning reproducibility at this time is approximately 10 µm or less. According to tests using a laser displacement meter, the positioning reproducibility of the axis stages without encoders regarding the movement of these units is less than ±5 µm in the *X* and *Y* directions. Actual positioning is carried out with the motorized stages (*X*-, *Y*- and *Z*-stages) mounted on each unit. A video of this operation is available in the supporting information. During this process, the relative positions between the SC and each axis of the diffractometer are calculated, and the position of the SC is automatically adjusted by the *X*-, *Y*- and *Z*-stages mounted on the SC.

Downstream of the diffractometer, an area detector unit is equipped with a long linear guide rail of approximately 1500 mm in the *Y* direction. This unit includes motorized stages that allow the detector to be freely adjusted in the *XYZ* directions. An FPD is currently installed (XRD1611, Varex Imaging) that is based on an amorphous Si substrate and a CsI:Tl scintillator, as shown in Fig. 3 ▸(*c*). Upstream of the diffractometer, a temperature control unit is installed to control the temperature of the capillary sample. The unit is furnished with both a Cryostream nitro­gen gas blower and a hot-nitro­gen gas blower, each installed on the *X*-, *Y*- and *Z*-stages. The switching of the blowers is automated and the blowers can be inserted at the sample position, enabling temperature control ranging from 90 K to 1100 K. In the case of *in situ* measurements, the unit is designed to retreat automatically up to 1000 mm upstream to provide substantial sample space.

### 2.4. Sample environments

To provide versatility in powder diffraction experiments, measurement systems under a variety of sample conditions, including temperature, time, pressure and gas atmosphere, are available. The sample environments for powder diffraction on BL13XU are coordinated with the system on BL02B2, aiming for commonality in equipment such that various devices can be used interchangeably between the beamlines. Frequently used Cryostream nitro­gen and hot-nitro­gen gas blowers are permanently installed on both beamlines and can be controlled in the ranges of 90 K to 473 K and 300 K to 1100 K, respectively. By combining these blowing devices with the robotic SC, automatic measurements across a wide range of temperatures are possible for up to 100 samples. Several temperature control equipment options are available: (i) an electric furnace (HTK1200, Anton Paar) capable of heating capillary samples up to 1473 K at a maximum rate of 50 K min^−1^, (ii) a closed-cycle type cryostat, which is a customized modification of an HE05 (ULVAC Cryogenics, Inc.), that allows measurements at temperatures ranging from 5 K to 400 K with the capillary sample oscillating about the external ω axis of the EG, and (iii) a Linkam stage (TS1500) designed for pellet and powder flat-plate samples, with a heating range of room temperature to 1673 K (Kobayashi *et al.*, 2023 ▸) at a maximum rate of 200 K min^−1^.

In addition to these temperature control systems, a remote gas- and vapour-pressure control (RGVPC) system (Kawaguchi *et al.*, 2020 ▸) is available. This system facilitates measurements in noncorrosive gas atmospheres ranging from 1 Pa to 1000 kPa in absolute pressure. Moreover, gas pressure control can be synchronized with the 2D CdTe detectors, enabling time-resolved powder diffraction in a gas atmosphere. Previously, the ability to spin the capillary was limited due to the stainless steel tube used to introduce the gas into the sample. However, more uniform Debye–Scherrer rings can now be achieved under various gas atmospheres. The cell for capillary samples under gas atmospheres has been updated. Notably, the capillary spinner cell can control the gas pressure inside the capillary while continuously rotating at speeds of up to 500 rpm (Kawaguchi *et al.*, 2021 ▸). The high-speed spinner mainly consists of a gas cell to hold the glass capillary with double O-rings, a contactless magnetic fluid seal, a brushless motor and bearings. Additionally, the spinner is equipped with translation and tilt stages for aligning the glass capillary sample. This device can be installed on the large sample table. Commissioning is also in progress for other sample environments, including a high-speed heating (∼1000 K min^−1^) furnace that can reach temperatures up to 1973 K using infrared heating, and a high-pressure cell employing a hydro­static cell for achieving pressures up to 400 MPa. These additions are anticipated to be available soon.

## 3. Data acquisition and performance

### 3.1. Scan modes using the six sets of 2D detectors

Powder diffraction patterns were collected using the six sets of 2D CdTe detectors in three primary measurement modes: standard, single-shot and high-resolution scan modes. The distinctions between these scan modes originate from the placement of the detectors on the 2θ axis and the sample-to-detector distance, as described in subsequent sections. These scanning methods are summarized in Table 1 ▸, and illustrative photographs are provided in Fig. S1 of the supporting information. Notably, for detector sensitivity correction, flat-field images for each X-ray energy are carefully applied. We also employ a count rate correction, and all the data presented in this paper have been corrected. The installation of the detectors is mechanically adjusted using dummy detectors and a coordinate measuring machine. After that, the detectors are actually mounted, each module is set at the same 2θ position (*e.g.* 10°) by moving the modules to this 2θ position at different sample-to-detector distances, and the data of the standard sample are measured. For the dataset, the geometric corrections for the 2D CdTe detectors, such as the calibrated distance, point of normal incidence and tilt parameter, are performed using the NIST standard sample LaB_6_ 660c and the *pyFAI* library (Kieffer *et al.*, 2020 ▸). This correction and flat-field correction allow the data to be neatly stitched without applying coefficients to each detector. The 2D diffraction images measured using the 2D CdTe detectors are integrated into 1D data using an in-house Python script that incorporates the *pyFAI* library. During this conversion, inactive pixels and several pixels between chips within the detector are masked, and comprehensive data processing is conducted. Ultimately, the data obtained from the six sets of detectors are merged into 1D data through a data merging process developed on the beamline. These operations are automatically processed on a server along with the measurements, enabling attainment of the final 1D data as the intensity data for continuous 2θ values.

#### 3.1.1. Standard scan

The standard scan involves measurements at four 2θ positions using the six sets of 2D CdTe detectors, covering a 2θ range from 0.6° to 78°. The measurements at these four positions are used to bridge the 2θ gaps between detectors and fill in the gaps within each detector. With a sample-to-detector distance of 630.24 mm, the angular resolution is approximately 0.005° per pixel, enabling coverage of approximately 7.9° in 2θ for each detector. Consequently, when detectors are spaced at 12.5° intervals, a gap of approximately 4.6° in 2θ is present. Hence, data acquisition at two positions is necessary to cover this gap. Additionally, the LAMBDA 750k detector consists of two sets of six Medipix3 chips. Between the sets of 3 × 2 and 3 × 2 chips (one chip size: 256 × 256 pixels), an inactive gap of ten pixels is present. Therefore, data acquisition from two locations where the 2θ position is slightly moved (*e.g.* by 0.64°, which is equivalent to 128 pixels from 0.005° × 128 pixels) is required. Thus, the whole powder diffraction data without gaps can be measured at 2θ = 0, 0.64, 6.25 and 6.89°. When the exposure time is set at 1 s, a whole powder pattern can be measured in approximately 24 s (1 s × 4 positions + an additional 20 s). The additional time of 20 s arises from idle periods due to mechanical movements of components such as the 2θ axis and the shutter. This technique is suited to high-throughput collection of powder diffraction patterns up to high-*Q* regions and is designed for crystal structure analysis, including Rietveld refinement and PDF analysis. The diffraction data obtained from standard scans are suitable for static structural investigation, and this type of scan is not recommended if there is a change in the sample conditions within a short period, *e.g.* less than a minute. The utilization of X-rays with an energy of 35 keV can achieve a maximum *Q* (*Q*_max_) of 20 Å^−1^. At 60 keV, this value surpasses 35 Å^−1^. An alternative for high-*Q* data collection is to start standard scans from high angles, which facilitates measurements up to a 2θ of 120°. Thus, collecting diffraction data up to *Q*_max_ = 30 Å^−1^ becomes feasible with a 35 keV X-ray energy by combining standard scans at low and high 2θ angles.

#### 3.1.2. Single-step scan

**Figure 4:**
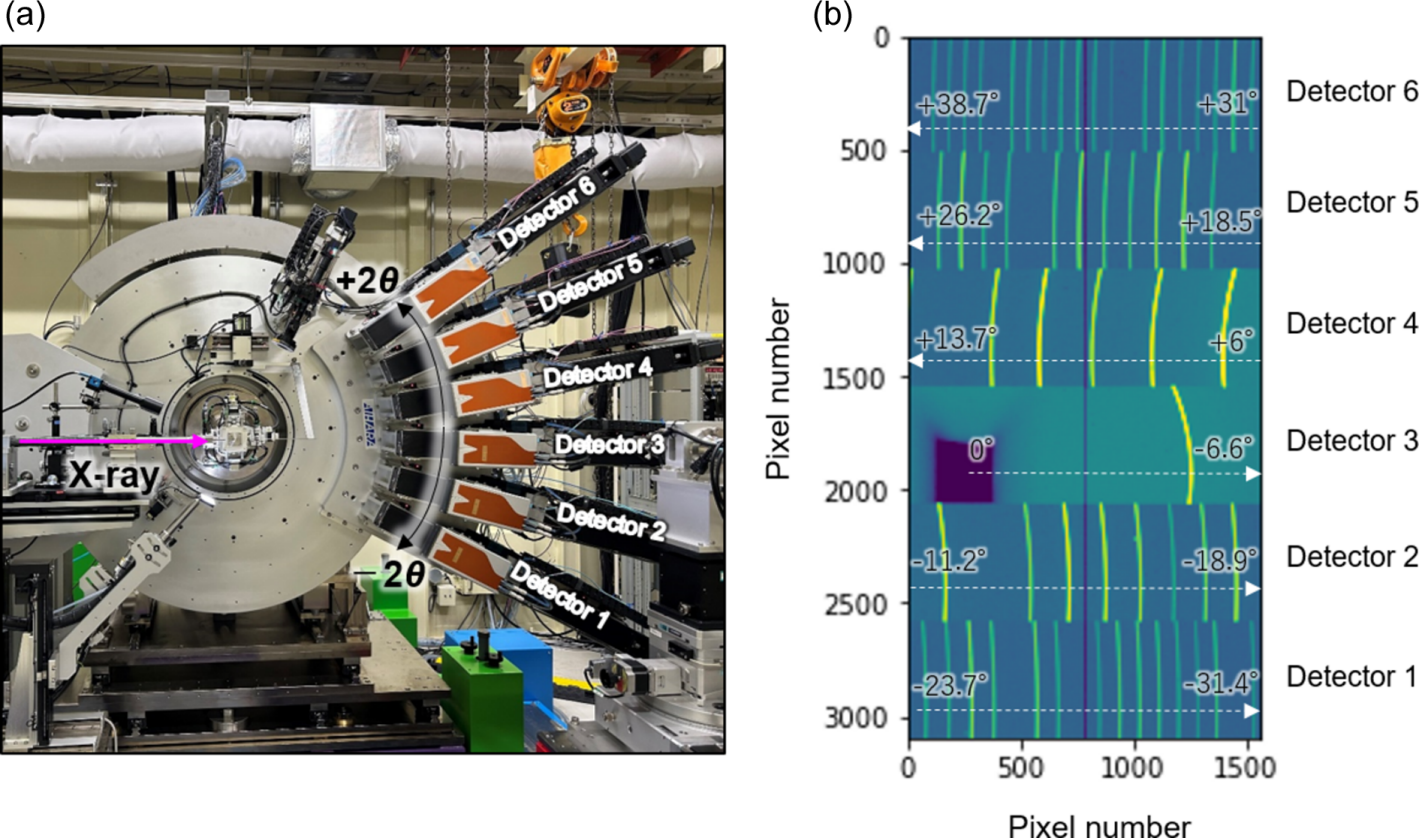
(*a*) A photograph of the powder diffractometer in the single-step mode. (*b*) Two-dimensional diffraction images taken by the six sets of 2D CdTe detectors in the single-step mode.

For rapid *in situ* powder diffraction and time-resolved experiments, the single-step mode provides significant advantages, as it enables the collection of the whole powder pattern without necessitating adjustment of the detector positions along the 2θ axis. The sample-to-detector distance in the single-step mode is set to the same distance as in the standard scan, and the detectors are asymmetrically set in the positive and negative 2θ directions, as shown in Fig. 4 ▸(*a*). This configuration is similar to that of previously developed methods (Katsuya *et al.*, 2016 ▸). Fig. 4 ▸(*b*) shows 2D diffraction images measured in this mode using the 2D CdTe detectors. In the figure, the 2θ range measured by Detector 3 is from 0° to −6.6°, and the adjacent Detector 2 can measure from −11.2° to −18.9°. At this time, the insensitive area from −6.6° to −11.2° is covered by Detector 4, which can measure from 6° to 13.7°. This asymmetric setup enables measurements up to 38° to be performed in a single shot. At 60 keV, the *Q* value reaches approximately 20 Å^−1^. Coupled with the 2 kHz high-speed imaging of the LAMBDA 750k detector in 12-bit mode, time-resolved measurements can be performed on the millisecond time scale under dynamic changes in the sample environment, including temperature, time and pressure variations. Transitioning between the standard and single-step modes is straightforward, only requiring more 2θ-axis movement.

#### 3.1.3. High-resolution mode

The high-resolution mode is designed to capture a whole powder pattern with the highest angular resolution achievable using the 2D CdTe detectors on BL13XU. In this mode, the sample-to-detector distance is extended to 1050.4 mm, which is greater than that in the standard mode. This configuration yields an angular resolution of 0.003° per pixel. The detector distance is automatically adjusted by electric motors simultaneously operating along six axes (refer to Video S1 of the supporting information). Each detector covers an approximate range of 4.7° in the 2θ region. As a result of this configuration, the gaps between detectors are more pronounced than those in the standard mode. To address these gaps, data acquisitions are conducted at three distinct 2θ positions. Moreover, similar to the standard mode, double acquisitions are performed to compensate for the inherent gaps within the detectors. Consequently, a total of six acquisitions are needed to produce a comprehensive powder pattern.

### 3.2. Area detector mode

Although the temporal resolution, measured in the 2θ range, and the statistical accuracy are less than those of a scan performed with the 2D CdTe detectors, an FPD (XRD1611) can be used as an option instead of the 2D CdTe detectors. This detector is primarily used for experiments that require a full Debye–Scherrer ring, such as texture analysis. Both *in situ*X-ray diffraction and PDF measurements can be conducted using this detector. For the former, the detector is arranged such that it can be positioned more centrally with respect to the beam. PDF measurements are often conducted using an offset arrangement, with the beam centre at the edge of the detector, to enable the acquisition of measurements over a wide 2θ range. The distance from the sample is adjustable from 350 to 1800 mm, and diffraction patterns up to 2θ = 40–50° can be recorded, depending on the detector position. Hence, when using X-rays with an energy of 70 keV, *Q*_max_ reaches ∼30 Å^−1^. Before being used, the geometric parameters of the detector, such as the distance from the sample and the rotation angles, are calibrated by measuring a NIST standard sample and refining the distances and rotation angles using the *pyFAI* library. Note that the wavelength has already been calibrated using a 2D CdTe detector scan. Additionally, prior to use, the background noise is subtracted from the collected data and the dark data are recorded. Typically, the detector captures multiple 1 s exposures, which are subsequently summed. However, faster measurements on the order of hertz are also feasible.

### 3.3. Performance

**Figure 5:**
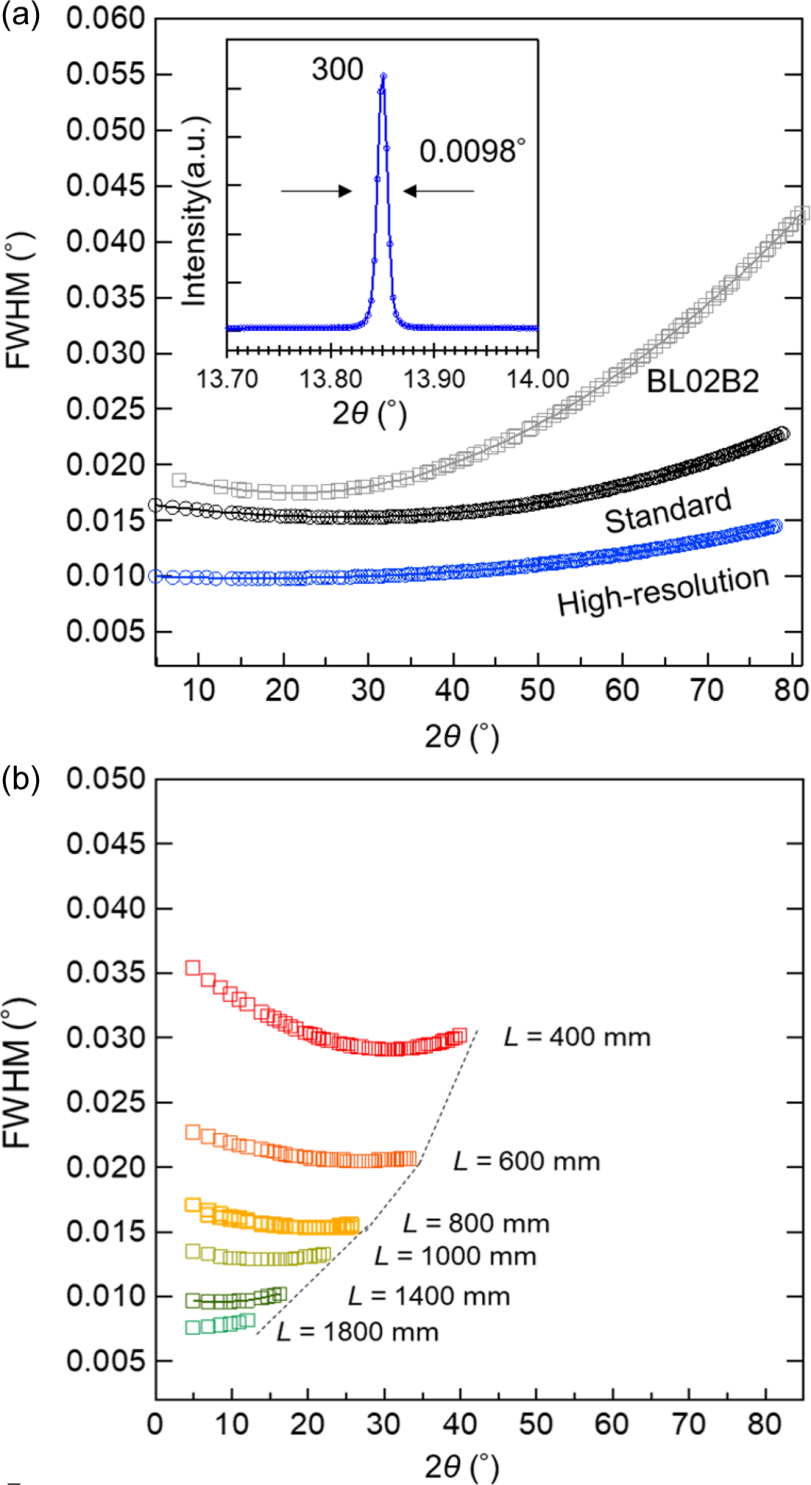
The FWHM as a function of 2θ for LaB_6_ powder. (*a*) Standard and high-resolution modes using the 2D CdTe detectors. For the BL02B2 data, the FWHM dependence was calculated from NIST Si 640C data. The inset shows a magnification of the 300 diffraction peak collected using the high-resolution mode. (*b*) Data from the FPD with various sample-to-detector distances (*L*). The LaB_6_ powder sample was loaded into a Lindeman glass capillary with a diameter of 0.2 mm. These data were collected using 35 keV X-rays.

The FWHM as a function of the 2θ value of the recorded diffraction peaks of NIST LaB_6_ 660c powder is shown in Fig. 5 ▸(*a*). The incident X-ray energy was set at 35 keV. The FWHM curves were calculated using the *JANA2006* software (Petříček *et al.*, 2014 ▸). All diffraction peaks were fitted by applying the Thompson–Cox–Hastings pseudo-Voigt function. The inset shows the observed powder diffraction profile of LaB_6_. The peak profiles remain almost symmetric across the entire 2θ region. In the high-resolution mode, the FWHM is approximately 0.01 to 0.015° in the full 2θ range. The scan data on BL13XU were obtained by azimuthally integrating 2D data into 1D data using an in-house Python script that incorporates the *pyFAI* library, and this integration reduces the axial divergence effect. Fig. S2 shows the diffraction peak of NIST LaB_6_ 660c powder for a capillary diameter of 0.2 mm in the lower 2θ region. Even in the lower 2θ region, the peak profile is symmetric. However, the peak may be slightly asymmetric in the lower 2θ region for capillaries with extremely small diameter, due to the axial divergence effect. Data measured with the MYTHEN detector (sample-to-detector distance of 477.47 mm) in the standard scan on BL02B2 are included for comparison. The standard scan shows an FWHM between 0.015° and 0.025°, indicating a higher angular resolution than that on BL02B2. This angular resolution is achieved while using a capillary with a diameter of 0.2 mm. However, with high-energy X-rays, capillaries with a diameter of 0.5 mm are sometimes used, and as the diameter of the capillary increases, the angular resolution is affected. The data in Fig. S3 were measured for various samples and diameters. As the diameter of the capillary increases, the width of the diffraction peaks becomes broader. In the Debye–Scherrer geometry, the dependence of the FWHM on the diffraction angle is affected by the X-ray optics, as well as instrumental and microstructural broadening (Bergamaschi *et al.*, 2010 ▸). This high angular resolution measurement is anticipated to be useful in the determination of complex structures such as metal–organic framework (MOF) compounds and in the study of small lattice distortions.

Fig. 5 ▸(*b*) shows the 2θ dependence of the FWHM measured for the LaB_6_ sample at various sample-to-FPD distances. The dependence of the FWHM for the FPD exhibits a slightly flatter shape than that for the 2D CdTe detectors, and the FWHM tends to be lower at higher angles when the sample-to-detector distance is reduced. This profile is further influenced by varying the sample-to-detector distance with respect to 2θ, aligning with similar experimental results (Dippel *et al.*, 2015 ▸). When the distance is smaller, the width of the diffraction peak becomes broader, but a large 2θ region can be measured. When the sample distance is more than 1400 mm, the angular resolution is less than 0.01°, but only a small 2θ range of ∼10° can be measured. These layouts can be modified based on experimental preferences, although measurements with close sample-to-detector distances in the range of 350–600 mm are typically performed to obtain a wide 2θ range.

**Figure 6:**
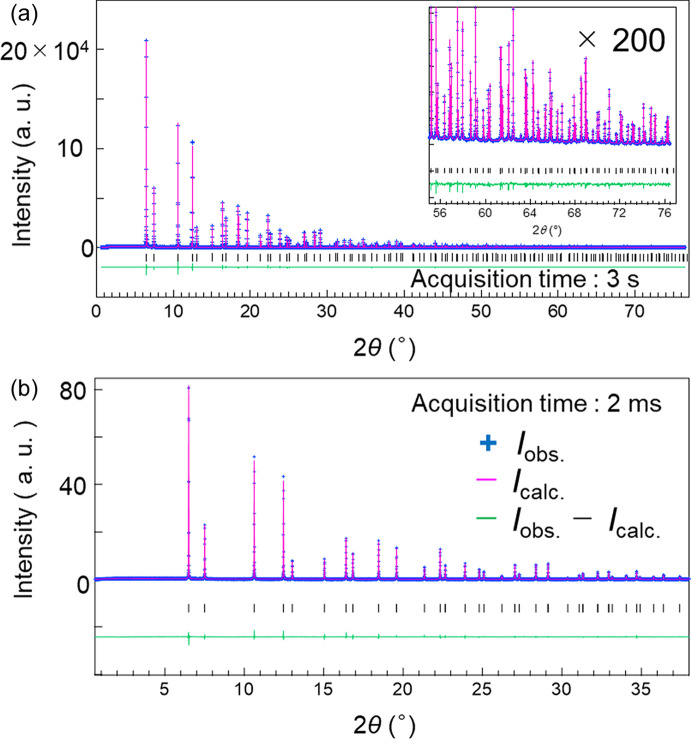
(*a*) Rietveld refinements of the NIST CeO_2_ powder data obtained in the standard scan mode. The inset shows an expanded view of the high-angle data. The powder was filled into a 0.2 mm diameter Lindeman glass capillary. (*b*) Rietveld refinements of the NIST CeO_2_ powder data obtained in the single-step scan mode. The markers and line show the measured (*I*_obs._) and calculated (*I*_calc._) data, respectively. The intensity difference curves (*I*_obs._ − *I*_calc._) are offset for clarity. All data were measured using an X-ray energy of 35 keV.

The powder diffraction data of NIST CeO_2_ 674b, obtained using the 2D CdTe detectors in the standard scan mode, were least-squares fitted based on the Rietveld method, as depicted in Fig. 6 ▸(*a*). The incident X-ray energy was set at 35 keV and the powder diffraction data, acquired with a duration of 3 s, were analysed using the *JANA2006* software program. The total measurement time for the data was approximately 32 s due to the use of the standard scan. The reliability indices of the Rietveld refinements were a weighted profile *R* factor (*R*_wp_) of 0.046, Bragg intensity *R* factor (*R*_B_) of 0.017 and goodness-of-fit (GOF) of 1.17. The isotropic atomic displacement parameters *U*_iso_ were 0.00308 (1) and 0.00563 (9) for the Ce and O atoms, respectively. The inset in Fig. 6 ▸(*a*) shows an expanded view of the high-angle data, highlighting the region around *Q* ≃ 20 Å^−1^. Even with an acquisition time of a few seconds, diffraction peaks were clearly observed in the high-*Q* region. Compared to the results from BL02B2, as shown in Fig. S4, the statistical quality of the high-angle data was enhanced due to the amplified X-ray flux from the ID source and the improved detection efficiency of the 2D CdTe detectors. Subsequently, we assessed the crystal structure analysis based on the powder diffraction data measured in milliseconds, and the results are shown in Fig. 6 ▸(*b*). The data for CeO_2_ were measured in the single-step mode utilizing 35 keV X-rays. The acquisition time was 2 ms. In the Rietveld refinement result, *R*_B_ was approximately 0.03 and the isotropic atomic displacement parameters were approximately the same as those obtained in the standard scan. This result indicates potential advancements in visualizing continuous changes in crystal structures on the millisecond time scale.

**Figure 7:**
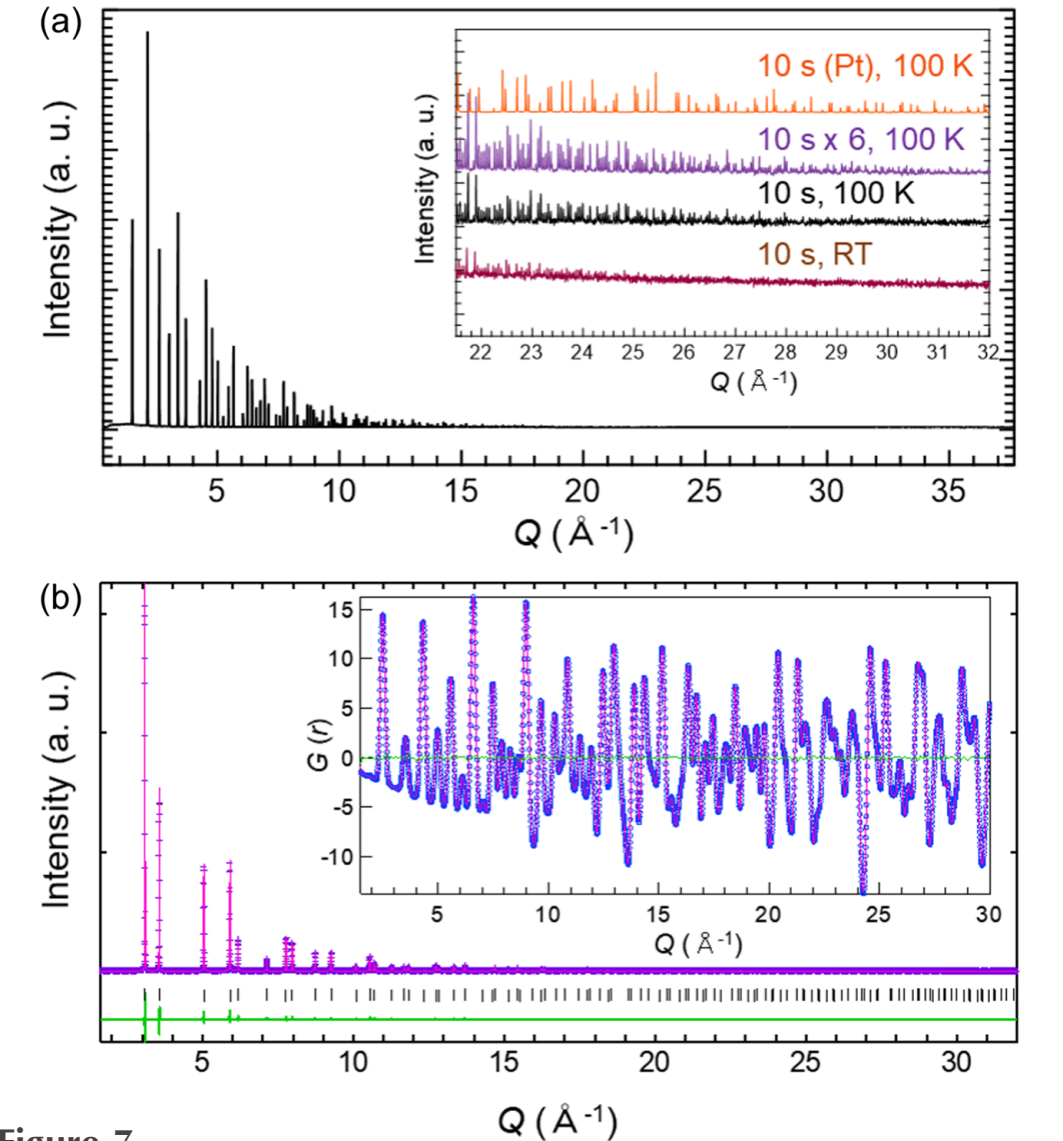
(*a*) The powder diffraction pattern of LaB_6_ at room temperature (RT) using an X-ray energy of 60 keV. The inset shows the diffraction patterns of the LaB_6_ and Pt powder samples in the high-*Q* region collected at room temperature and 100 K. (*b*) The Rietveld fitting result for the Ni powder sample. The inset shows the PDF fitting result obtained using *PDFgui*. The dotted plot shows the measured data and the line shows the calculated data.

Fig. 7 ▸(*a*) shows the standard scan data of NIST LaB_6_ at room temperature, measured using high-energy X-rays at 60 keV. The acquisition time was 10 s. To facilitate the observation of peaks in the high-*Q* region, the sample was cooled to 100 K using a Cryostream nitro­gen gas blower. As illustrated in the figure, diffraction data over *Q* = 30 Å^−1^ were obtained. The inset provides the data averaged over six repetitions of the same scan to compare the intensities at high angles. Detailed magnification at the high-*Q* region and whole powder diffraction data are shown in Fig. S5. By repeating the scans, weak peaks four to five orders of magnitude lower than the highest intensity peak were clearly detected, even near *Q* ≃ 30 Å^−1^. Additionally, for certain samples, distinct Bragg peaks can be observed up to the high-*Q* region exceeding 30 Å^−1^, even with an acquisition time of approximately 10 s. For instance, the platinum powder data at 100 K, shown in the same figure, reveal this capability. The data for CeO_2_ measured at 20, 25, 30, 35, 50, 60 and 72 keV are shown in Fig. S6. The measurements were performed in the standard scan mode, which shows how wide a *Q* region can be measured at each energy. Thus, this diffractometer facilitates measurements up to the high-*Q* region in a short amount of time.

The dataset acquired by the 2D CdTe detectors can be analysed using both Rietveld refinement and PDF analysis. Fig. 7 ▸(*b*) shows the standard scan data for the Ni powder obtained with 60 keV X-rays. Commercial Ni powder was filled into a 0.5 mm diameter Lindeman capillary. The acquisition time was 10 s. The collected data were fitted using the Rietveld method. After subtraction of the background intensity and data corrections, the data were normalized to obtain the Faber–Ziman total structure factor *S*(*Q*) (Faber & Ziman, 1965 ▸) using *Q*_max_ = 30 Å^−1^. Subsequently, the reduced PDF, *G*(*r*) (Billinge, 2008 ▸), was obtained. The Rietveld and PDF fits were obtained using *TOPAS* and *PDFgui* software, respectively (Coelho, 2018 ▸; Farrow *et al.*, 2007 ▸). For the PDF fitting, the observed and calculated profiles effectively aligned, resulting in reduced χ^2^ = 0.012 and *R*_w_ = 0.022. To explore the influence of the count time on the acceptable counting duration, data for the Ni powder were collected over intervals ranging from 1 to 60 s, as shown in Fig. S7. When the same fitting was performed for each dataset, the *R*_w_ value no longer improved above 10 s of data acquisition time. Therefore, for Ni powder, an acquisition time of 10 s is sufficient for PDF analysis. For crystalline materials that include nanoparticles, both Rietveld and PDF analyses can be performed. PDF analysis for multi-element nanoalloys using this powder diffractometer has already been reported (Minamihara *et al.*, 2023 ▸).

### 3.4. Demonstration

**Figure 8:**
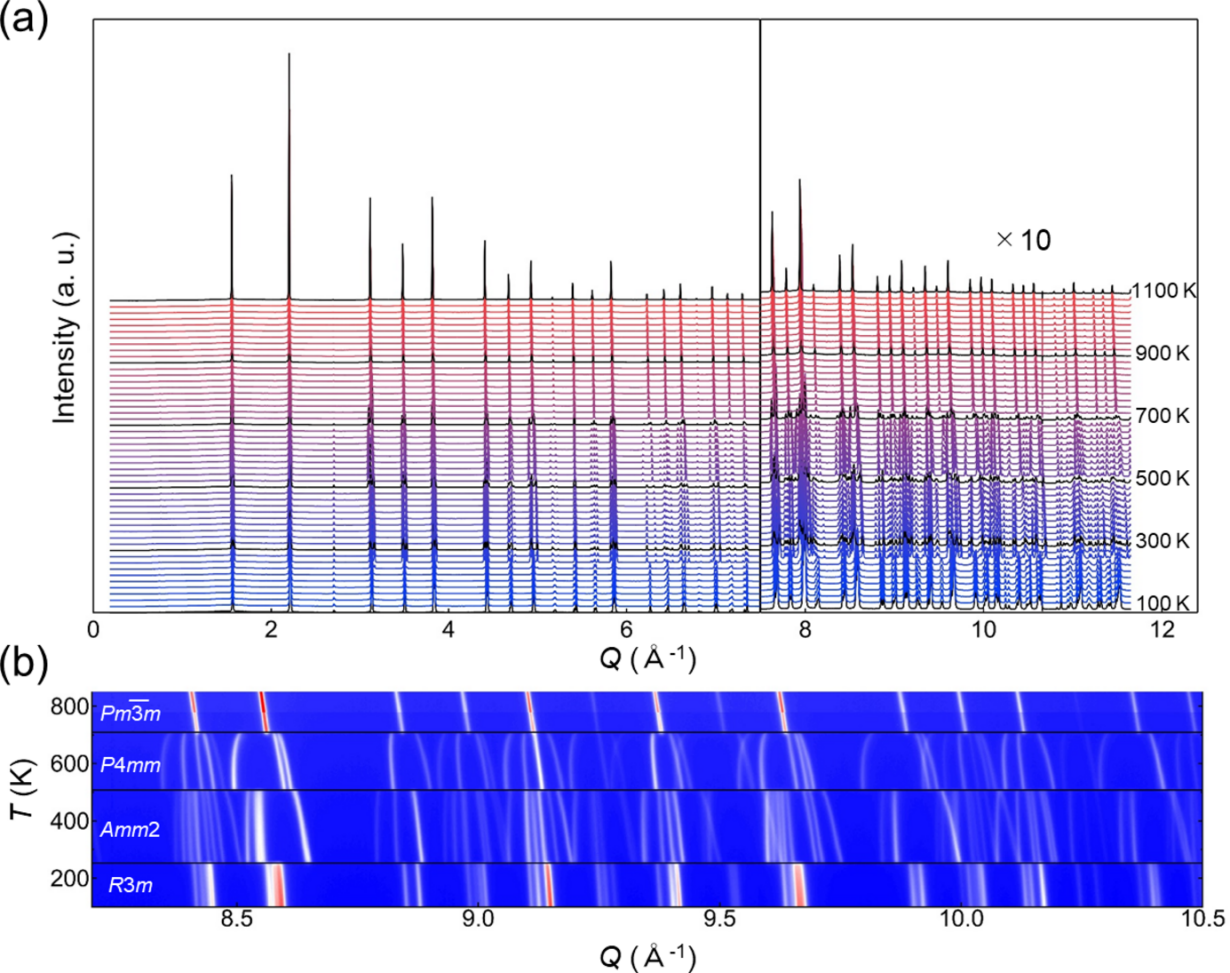
(*a*) The temperature dependence of the powder diffraction patterns of KNbO_3_ powders recorded in the single-step mode using an X-ray energy of 35 keV. (*b*) A 2D plot of the powder patterns.

Using the developed powder diffractometer system, we conducted quick *in situ* observations of successive phase transitions in the classical ferroelectric perovskite KNbO_3_. This demonstration was designed to show the ability to acquire diffraction patterns with a high signal-to-noise (S/N) ratio, even during rapid temperature changes. A commercial KNbO_3_ powder (Kojundo Chemical; >99%) sealed in a 0.3 mm diameter quartz capillary with Ar gas was used for the measurements. The incident X-ray energy was set at 35 keV and the powder pattern acquisition time was 1 s per frame using the 2D CdTe detectors in the single-step mode. The sample temperature was controlled from 100 K to 300 K using a Cryostream nitro­gen gas blower and from 300 K to 1100 K with a hot-nitro­gen gas blower. The temperature ramp rate was set at 80 K min^−1^. The temperature dependence of the diffraction patterns is displayed in Fig. 8 ▸(*a*). Remarkably, despite the short exposure time and high temperature ramp rate, powder diffraction patterns can be collected with a high S/N ratio, even up to the maximum measurable *Q* value of ∼11.6 Å^−1^. A selected portion of the powder diffraction intensity maps (2θ versus temperature) is shown in Fig. 8 ▸(*b*). The diffraction patterns exhibit drastic changes at 253 K, 506 K and 706 K; these changes correspond to the structural transitions from rhombohedral (*R*3*m*) to orthorhombic (*Amm*2), orthorhombic to tetragonal (*P*4*mm*), and tetragonal to cubic (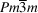) structures, respectively. These phase transition temperatures are consistent with previous reports (Kawamura *et al.*, 2013 ▸; Shirane *et al.*, 1954 ▸). Therefore, the sample was sufficiently heated, and structural changes were effectively detected even under rapid heating. Our developed diffractometer system is capable of performing *in situ* measurements across a broad temperature region in a condensed time frame of only 20 min.

**Figure 9:**
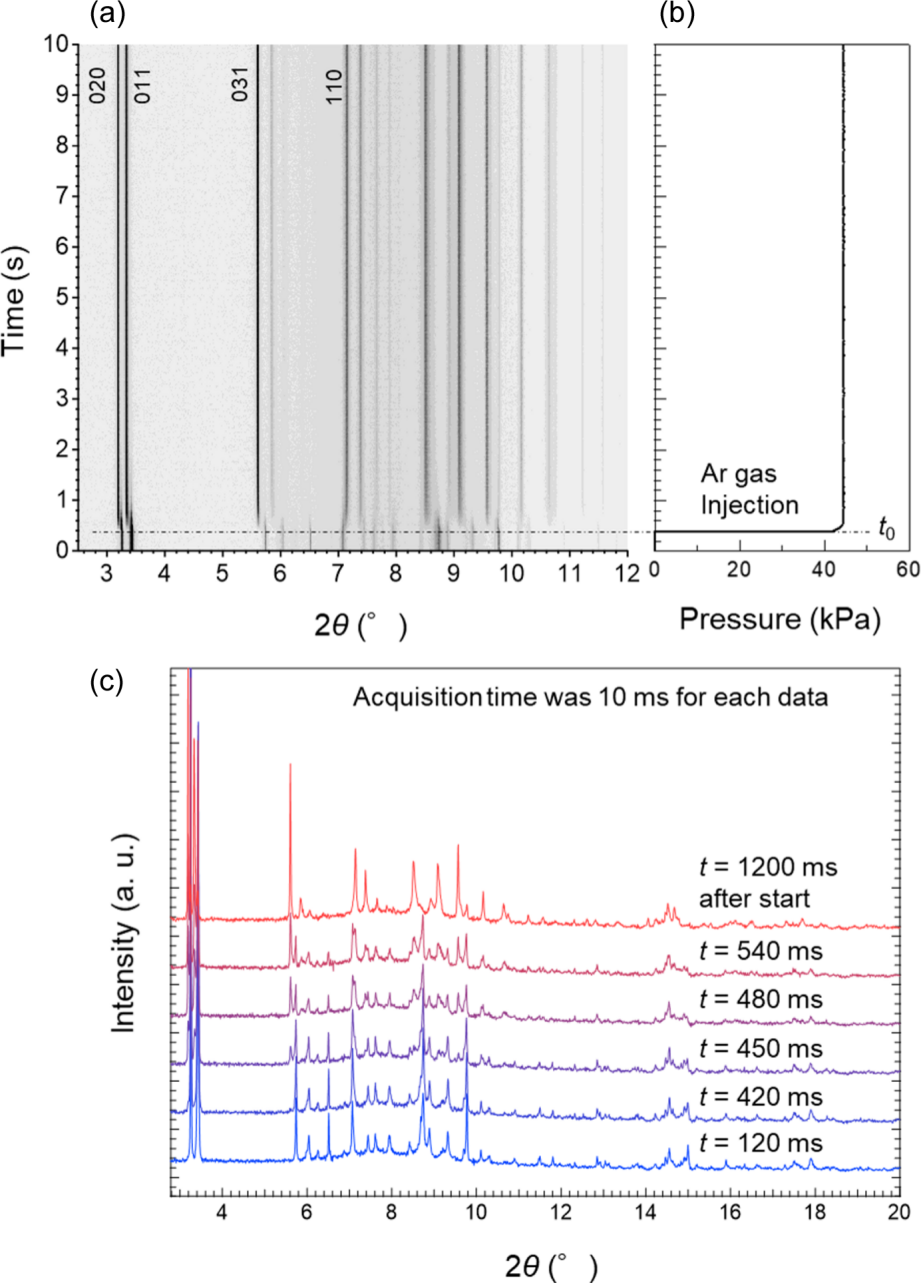
(*a*) A time-resolved powder diffraction intensity map of the CPL-1 sample at 100 K obtained in the gas-shot mode using an X-ray energy of 22 keV. (*b*) The time dependence of the Ar gas pressure. (*c*) The 1D powder diffraction patterns 120, 420, 450, 480, 540 and 1200 ms after the measurement started.

Further demonstrations show the ability to perform time-resolved experiments on the millisecond time scale under controlled gas pressure. The sample under investigation was a MOF complex, which was a nanoporous Cu coordination polymer, denoted CPL-1, formula [Cu_2_(pzdc)_2_(pyz)]*_n_* (pzdc = pyrazine-2,3-di­carboxyl­ate; pyz = pyrazine). This compound has a pillared layer structure containing 1D nanochannels with dimensions of 4.0 Å × 6.0 Å along the *a* axis between 2D sheets. The compound follows a typical physisorption process (Type I isotherm) and is known to adsorb various gases (Kitaura *et al.*, 2005 ▸). Utilizing the RGVPC system in conjunction with the capillary gas spinner cell and Cryostream nitro­gen gas blower, we achieved control over both the sample temperature and gas pressure. The CPL-1 powder sample was filled into a borosilicate glass capillary with a 0.5 mm diameter. The filled capillary was then positioned within the gas spinner cell. The incident X-ray energy was set to 22 keV. The ‘gas-shot’ mode instantly delivered predetermined amounts of gas to the sample; using this mode, we executed time-resolved powder diffraction assessments of the CPL-1 sample during the Ar gas adsorption process. Fig. 9 ▸(*a*) shows the time-dependent evolution of the powder diffraction intensity. Concurrently, Fig. 9 ▸(*b*) traces the temporal fluctuations of the Ar gas pressure. We continuously captured powder diffraction patterns at a frequency of 100 Hz, with an acquisition time of 10 ms for each whole powder pattern. At 0.4 s (*t*_0_) after the measurements were initiated, 44 kPa of Ar gas in the RGVPC system was automatically introduced into the sample in the glass capillary by opening the diaphragm-sealed valve near the gas cell. Fig. 9 ▸(*c*) shows the 1D powder diffraction patterns 120, 420, 450, 480, 540 and 1200 ms after the measurement started. A transformation in the 1D pattern signifying a crystal structure change from the desorption phase to the Ar gas adsorption phase is evident; additionally, the diffraction patterns of the desorption and Ar gas adsorption phases correspond to those in the literature (Ashitani *et al.*, 2023 ▸). Previously, with the conventional instrument on BL02B2, the boundaries of time-resolved measurements were restricted to a few hundred milliseconds due to the lower X-ray flux and detector efficiency for high-energy X-rays than those on BL13XU; this resulted in the acquisition of only sparse diffraction peaks. In contrast, when using the updated powder diffraction system on BL13XU, distinct diffraction peaks up to high 2θ regions are discernible, featuring a temporal resolution that is superior by several tens of times. Despite the relatively low diffraction intensity from organic materials such as MOFs, sufficient data for analysing structural changes were successfully obtained using a 10 ms acquisition time. For samples such as metals and oxides, which exhibit significantly higher diffraction intensities, rapid measurements of the order of kilohertz are feasible. In recent years, groundbreaking insights have been obtained from time-resolved experiments under controlled gas atmospheres [as shown by Hiraide *et al.* (2020 ▸), Yamamoto *et al.* (2023 ▸) and Sakanaka *et al.* (2023 ▸)]. The advanced temporal resolution of this system, combined with expansive *Q*-range measurements facilitated by high-energy X-rays, has advancement potential in deciphering the mechanisms of adsorption and reaction processes by conducting time-resolved structural analysis.

## 4. Conclusion

We have designed and implemented a new high-resolution powder diffractometer in the third experimental hutch of the ID beamline BL13XU. This diffractometer is equipped with six sets of 2D CdTe (LAMBDA 750k) detectors and an automation system. Utilizing the six sets of 2D CdTe detectors, we have developed three distinct scan modes: standard, single-step and high-resolution. The standard scan can acquire data with a high-*Q* value exceeding 30 Å^−1^ at 35 keV within several tens of seconds. The high-resolution mode enables the acquisition of measurements with an FWHM of less than 0.01° at low 2θ angles. The single-step mode, in which multiple detectors are asymmetrically positioned in the positive and negative 2θ directions, enables continuous data acquisition on the millisecond time scale. While its angular range is narrower than that of the standard scan, a sufficient *Q* region can still be measured using high-energy X-rays, such as 35 keV and 60 keV. The capability of our developed system is demonstrated by Rietveld and PDF analyses of standard samples, observations of structural phase transitions under fast temperature variation conditions, and time-resolved experiments under gas adsorption processes. In addition to the 2D CdTe detectors, a large FPD is available but it has a lower temporal resolution (∼1 s), a more limited 2θ range and a lower dynamic range compared to the scan modes using the 2D CdTe detectors. The FPD can be freely positioned on motorized *XYZ* stages, enables the observation of full Debye–Scherrer rings, and is suitable for detecting the preferred orientation, strain and large crystal grains in samples.

An automated equipment switching system was developed to simplify the setup changes around the diffractometer and ensure consistent reinstallation. This system features a robotic SC for 100 capillary samples, a large sample table with an 800 mm × 600 mm surface for heavy equipment and an EG for oscillating heavy equipment. This development not only automates the switching between *in situ* and automatic measurements but also enables various *in situ*/*operando* measurements by providing a large sample space. The beamline setup for the sample environments is aligned with that of BL02B2, ensuring equipment compatibility. A fully automatic system using a combination of temperature control nitro­gen gas blowers and a robotic SC allows automatic measurements in the temperature range from 90 K to 1100 K in a short time. The RGVPC system enables the acquisition of measurements in diverse gas atmospheres and has been synchronized with the 2D CdTe detectors.

In the future, we will actively continue to develop the measurement system to realize a wide variety of *in situ*/*operando* observations and intend to develop scan methods that take advantage of the variable sample-to-detector distance. Our powder diffractometer system, which is designed to obtain high throughput and high resolution, can be used to enhance various scientific studies, including phase identification and structural analysis using Rietveld refinement and PDF analysis. Additionally, observations of chemical reactions, gas storage and catalysis with millisecond precision are anticipated to be obtained, potentially revealing snapshots of crystal structures under irreversible processes.

## Supplementary Material

Figures S1 to S7. DOI: 10.1107/S1600577524003539/ok5110sup1.pdf

The automatic equipment switching system. DOI: 10.1107/S1600577524003539/ok5110sup2.mp4

## Figures and Tables

**Table 1:** Specifications of the scan modes using the six sets of 2D CdTe (LAMBDA 750k) detectors X-rays with energies ranging from 16–72 keV are available.

Parameter	Standard mode	Single-step mode	High-resolution mode
2θ range (°)	0.6–77 (optionally up to 120)	0.6–38	0.6–77
Sample-to-detector distance (mm)	630.24	630.24	1050.4
Δ2θ (°)	0.005	0.005	0.003
Number of acquisitions needed for a whole powder pattern	4	1	6
Interval time between scans (s)	20	0	30
