# Supplementary material for: High-throughput and high-resolution powder X-ray diffractometer consisting of six sets of 2D CdTe detectors with variable sample-to-detector distance and innovative automation system
Source: J Synchrotron Radiat. 2024 Jun 20;31(Pt 4):955–67. doi: 10.1107/S1600577524003539 (PMC11226175; doi:10.1107/S1600577524003539)
Supplement: Supplementary file 1 [file s-31-00955-sup1.pdf]

JOURNAL OF SYNCHROTRON RADIATION

Volume 31 (2024)

Supporting information for article:

High-throughput and high-resolution powder X-ray diffractometer consisting of six sets of 2D CdTe detectors with variable sample-to-detector distance and innovative automation system

Shogo Kawaguchi, Shintaro Kobayashi, Hiroki Yamada, Hirotaka Ashitani, Michitaka Takemoto, Yasuhiko Imai, Takaki Hatsui, Kunihisa Sugimoto and Osami Sakata

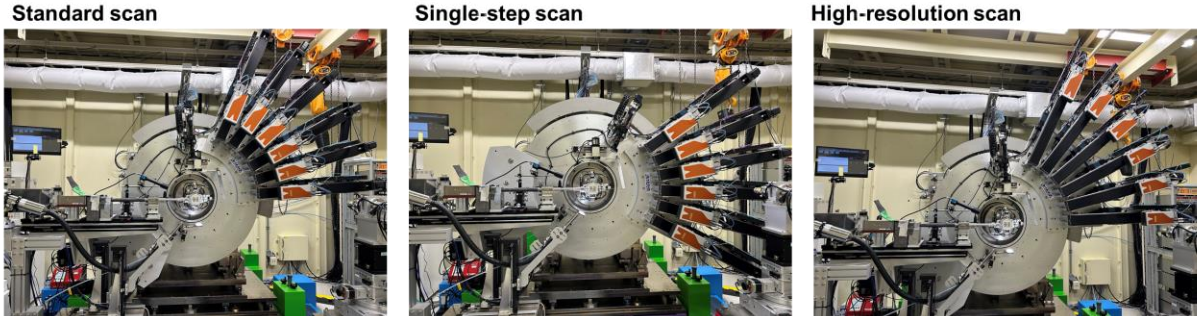

Figure S1 Photographs of the powder diffractometer in the standard, single-step, and high-resolution scan modes.

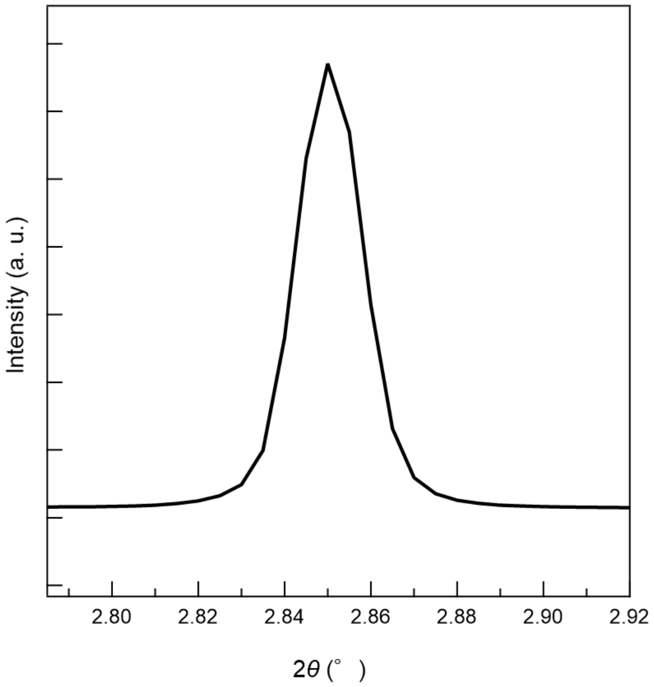

Figure S2 LaB_6 powder 100 diffraction peak measured using an X-ray energy of 60 keV. The powder sample was loaded in a Lindeman glass capillary with a diameter of 0.2 mm.

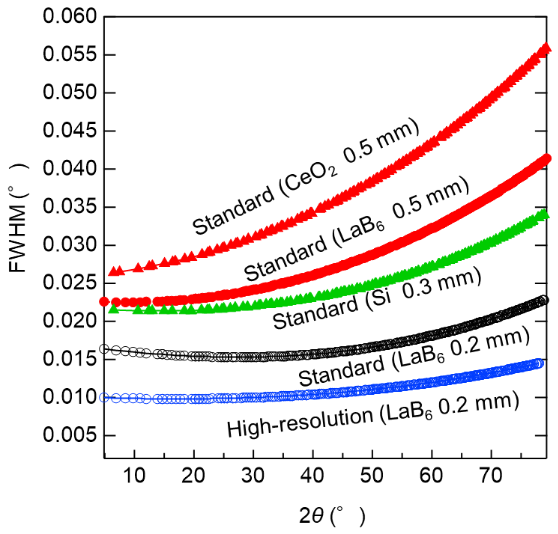

Figure S3 FWHM as a function of 2θ for the LaB_6 powder when using capillaries with diameters of 0.2 and 0.5 mm, NIST Si 640c when using a capillary with a diameter of 0.3 mm, and NIST CeO_2 674b when using a capillary with a diameter of 0.5 mm. The X-ray energy was set to 35 keV.

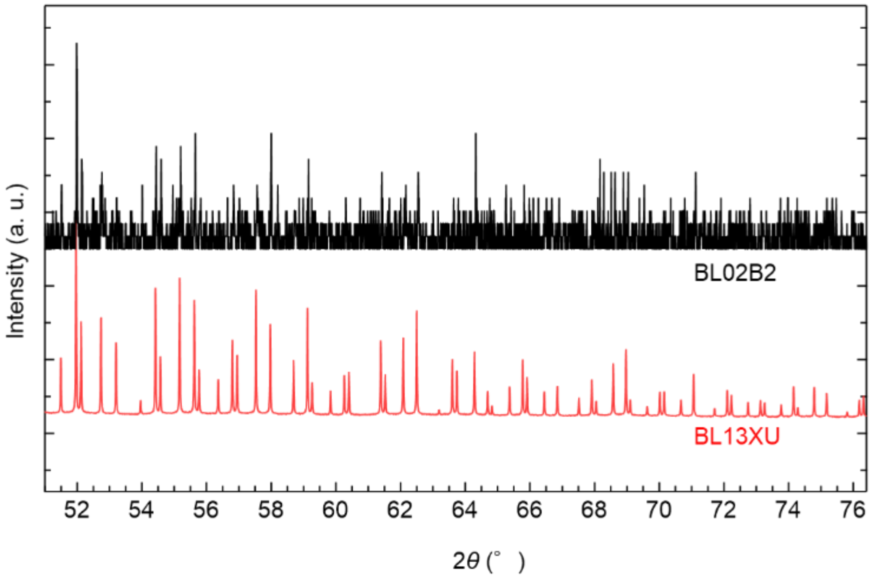

Figure S4 Powder diffraction patterns of the CeO_2 powder measured at BL13XU (red) and BL02B2 (black). For both sets of data, the X-ray energy was set to 35 keV, and the acquisition time was 1 s. For comparison, both data sets with the highest peaks are normalized to the same intensity. The BL02B2 data is also offset in terms of intensity for clarity.

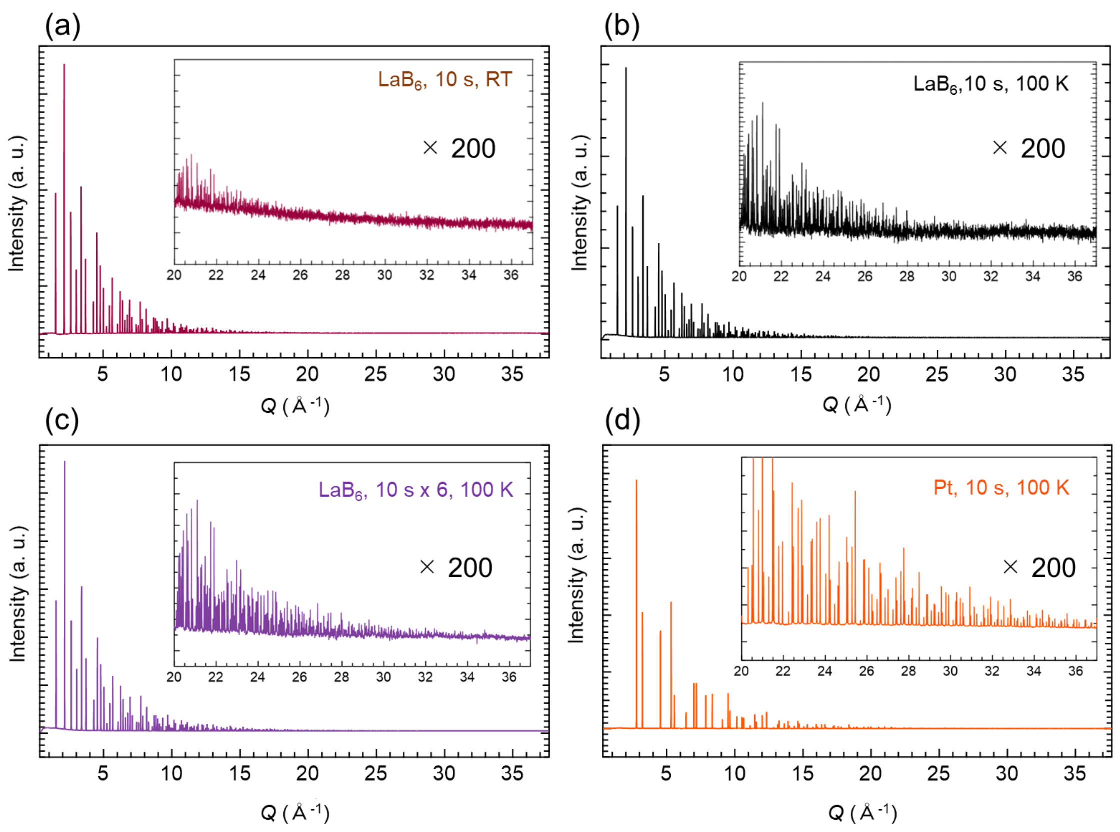

Figure S5 Powder diffraction pattern of LaB_6 at (a) room temperature (RT) and (b) 100 K. (c) The data were obtained for LaB_6 at 100K by repeating the standard scans with an acquisition time of 10 s. (d) The data of Pt powder samples at 100 K. The X-ray energy was set to 60 keV.

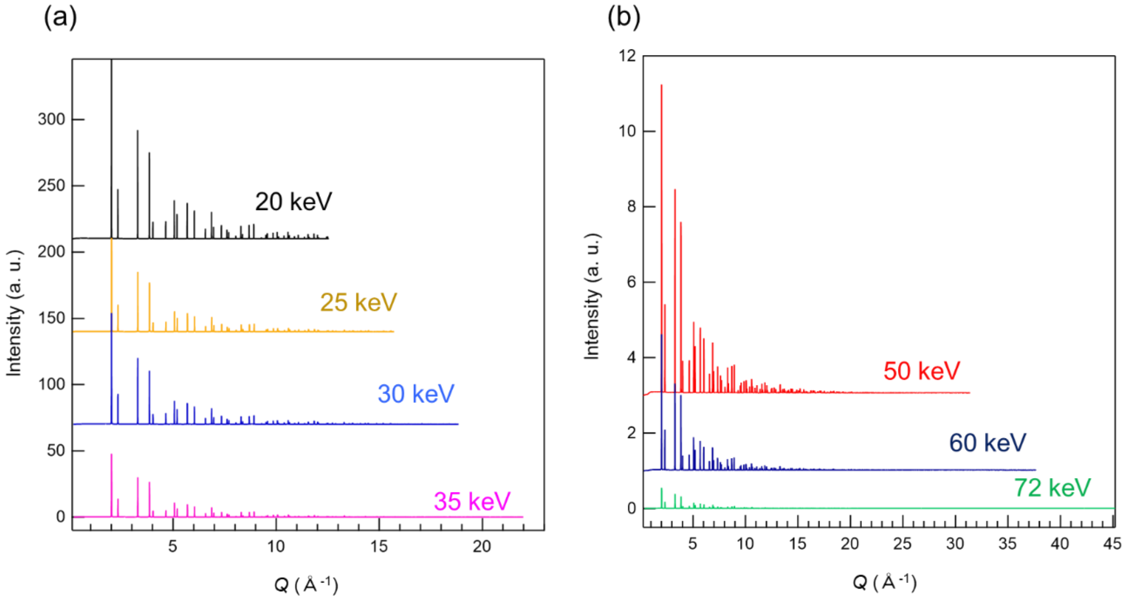

Figure S6 Powder diffraction patterns of the CeO_2 powder for a diameter of 0.2 mm measured in the standard scan mode. For all data, the acquisition time was 1 s.

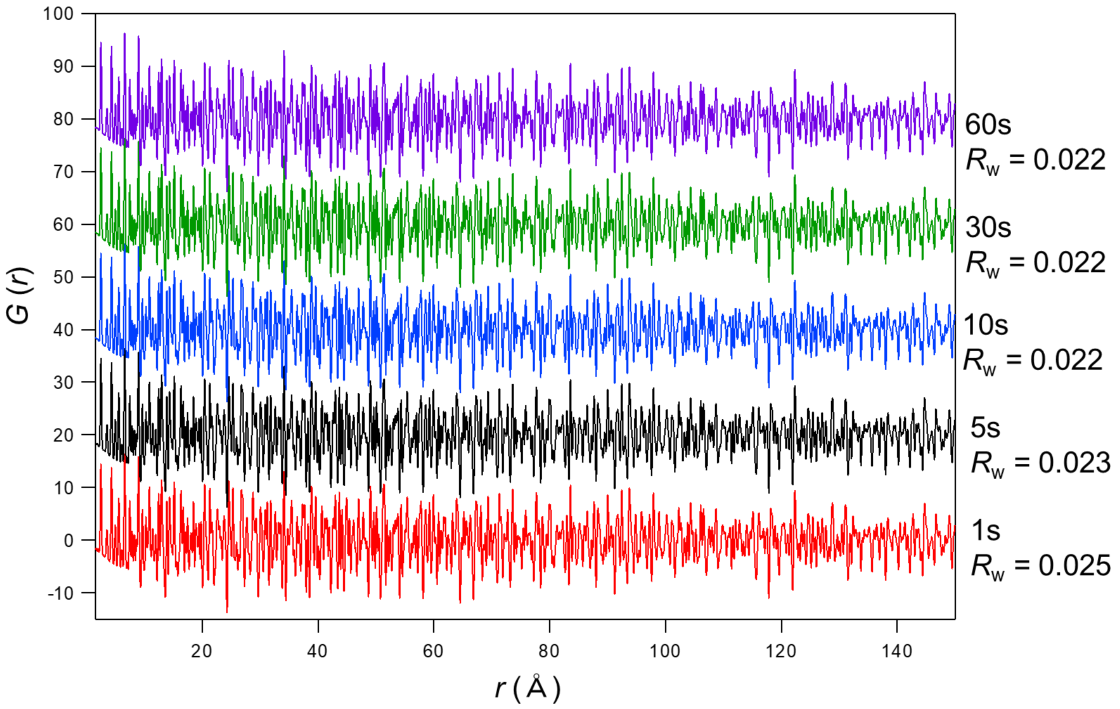

Figure S7 Pair distribution functions obtained from the data collected with different acquisition times. Data are offset by +20 each for comparison.
